# Evaluation of a Chinese herbal supplement on equine squamous gastric disease and gastric fluid pH in mares

**DOI:** 10.1111/jvim.15603

**Published:** 2019-08-23

**Authors:** Amelia S. Munsterman, Ana Sofia Dias Moreira, Fernando J. Marqués

**Affiliations:** ^1^ Department of Surgical Sciences, School of Veterinary Medicine University of Wisconsin Madison Wisconsin; ^2^ Department of Medical Sciences, School of Veterinary Medicine University of Wisconsin Madison Wisconsin

**Keywords:** Chinese herbal, horse, Wei Le San

## Abstract

**Background:**

Wei Le San (WLS) is a Chinese herbal formula comprised of 9 herbs selected for their putative anti‐inflammatory effects.

**Objectives:**

To evaluate the effects of WLS administration in horses with nonglandular gastric ulcers.

**Animals:**

Ten mixed breed mares (aged 7‐21 years, 401‐567 kg body weight).

**Methods:**

Experimental design was a blinded, prospective, 2‐period crossover study. All horses received a placebo (25 mL dextrose‐based syrup; n = 10) and the treatment (WLS, 5 g in 25 mL dextrose‐based syrup; n = 10), administered twice daily. Horses underwent a 1‐week, alternating feed‐deprivation period to induce or worsen existing ulcers; treatment began on day 7. Gastroscopic examination was performed on d0, d6, and d35, with gastric fluid pH obtained on d6 and d35. Gastric ulcer scores assigned by 3 masked observers were averaged for each examination.

**Results:**

Ulcer number scores for horses treated with WLS (median = 0; range, 0‐4) was not different from the untreated controls (median = 0.5; range, 0‐4; *P* = .81) by the end of the treatment period. Ulcer severity score for treated horses (median = 0; range, 0‐1) was also unchanged compared to the control group (median = 0.5; range, 0‐1; *P* = .85). Gastric pH was not altered by either treatment, with a median of 2.1 (range, 1.9‐4.1) for the horses treated with WLS and 2.8 (range, 1.6‐7.2) in the untreated controls (*P* = .46).

**Conclusions and Clinical Importance:**

The experimental model used to induce gastric ulceration was unable to discern a difference between the herbal supplement and the placebo in normal horses.

**Abbreviations**
ESGD: equine squamous gastric disease
GEE: generalized estimated equations
WLS: Wei Le San

## 1 INTRODUCTION

Equine Gastric Ulcer Syndrome is a cause of morbidity in domesticated horses.^1^, ^2^, ^3^, ^4^ The incidence of gastric ulcers in the squamous, nonglandular region, defined as equine squamous gastric disease (ESGD) is higher than 80%.^3^, ^5^, ^6^, ^7^, ^8^, ^9^ Prevalence of ESGD is closely linked to the athletic occupation of the horse, with higher rates in racehorses and high level endurance horses, compared to sport horses and those with less active lifestyles.^4^, ^5^, ^6^, ^7^, ^8^, ^9^, ^10^, ^11^, ^12^ The development of ESGD is likely tied to intensive management techniques, including exercise routines, feeding schedules, and confinement in stalls.^1^, ^12^, ^13^

Treatment of ESGD centers on medications that reduce gastric acid secretion. These include proton pump inhibitors that inhibit the H^+^K^+^ATPase pump in the secretory membrane of the gastric parietal cell, or H_2_‐receptor antagonists that block the parietal cell receptor.^4^ The most commonly used treatment protocol for ESGD is to administer medications to maintain gastric pH above 4, and omeprazole is the current treatment of choice.^4^ Medical management of gastric ulceration in horses involves daily administration of these drugs for up to 28 days or more, which is a financial burden for owners of horses.^14^, ^15^

Recently, a Chinese herbal formulation, Wei Le San (WLS), has been recommended for the treatment of mild to moderate gastric ulceration in horses.^16^, ^17^, ^18^ This proprietary herbal formula is derived from a combination of 2 traditional herbal formulas: Xiao Yao San and Er Chen Tang. It contains 9 herbs selected for their putative individual abilities, in addition to their proposed synergistic effects on the gastrointestinal tract. A commercially manufactured preparation is available but has limited clinical evidence to support its use as a supplement in horses with gastric ulceration.^16^

The purpose of this study was to determine the efficacy of WLS for equine nonglandular gastric ulcers induced by an intermittent feeding regime. We hypothesized that the administration of WLS would significantly decrease the nonglandular gastric ulceration scores compared to the control treatment, without an alteration in the gastric fluid pH.

## 2 MATERIALS AND METHODS

All procedures performed on the horses in this study were evaluated and approved for ethical use of animals by the University of Wisconsin Institutional Animal Care and Use Committee (Protocol #V005953), and the protocol followed the NIH Guide for Care and Use of Laboratory Animals.

A total of 10 healthy, adult mares (7‐21 years of age; 400.9‐567.2 kg body weight) from the resident herd at the University of Wisconsin Charmany teaching facility were obtained as a convenience sample. A minimum sample size of 10 was determined using ordinal logistic regression for the differences in ulcer number scores, using an alpha of 0.05, power of 0.8, and a 2‐tail test.^19^ Mares were not screened based on endoscopic ulcer scores before the study. Breeds of horse included 3 American Quarter Horses, 3 Thoroughbreds, and 1 each of a warm blood, Paint, Tennessee walking horse, and Paso Fino. At the time of enrollment in the study, a physical examination was performed to exclude those with clinical disease. All horses were vaccinated for Eastern Equine Encephalitis, Western Equine Encephalitis, West Nile Virus Encephalitis, Tetanus, and Influenza, and an anthelmintic (ivermectin, 0.2 mg/kg, PO) was provided twice yearly as part of the routine health management program.

### 2.1 Experimental design

The experimental protocol was designed as a 2‐period, crossover design. Each horse completed the 5‐week protocol twice, once with herbal treatment and once with the placebo to serve as their own controls. The horses were ranked and paired by ulcer number and severity combined scores (Table 1),^14^, ^20^, ^21^ after the first week of feed deprivation in period 1, and the pairs then randomly allocated by coin flip to 1 of 2 groups for the remainder of the study. During the study period, the horses were housed in a dry lot and fed a free choice, native grass hay ration. The treatment period was 5 weeks (35 days) in duration, where the first week consisted of a modified intermittent feed‐deprivation protocol designed to induce nonglandular gastric ulceration or worsen existing ulcers.^22^ During the first week of each 5‐week period, the horses were deprived of feed for 24 hours, then fed their normal grass hay ration free choice for 24 hours, until a total of 96 hours of cumulative feed deprivation were achieved. Throughout the protocol, the horses had free access to water, except for 4 hours immediately before gastroscopy.

**Table 1:** Number and severity scoring system (from MacAllister et al^17^)

**Gastric squamous ulcer number score**	
0	No ulcers observed
1	1‐2 localized ulcers
2	3‐5 localized ulcers
3	6‐10 localized ulcers
4	>10 ulcers or diffuse ulcers
**Gastric squamous mucosal ulcer severity score**	
0	No ulcers
1	Ulcer appears subjectively superficial
2	Ulcer appears to involve deeper structures, than superficial ulcers scored as 1
3	Multiple squamous ulcers present with variable severity (graded 1or 2)
4	Ulcers are graded 2 or 3 and have an active appearance, with hyperemia, a dark crater, or both
5	Ulcers are similar to those in 4, with active hemorrhage or an adherent blood clot

The treatment (placebo or herbal supplement) was administered after this feed deprivation period, starting on day 7, for a total of 4 weeks. The control group received 25 mL of an artificially flavored corn syrup‐based solution (ALDI, Inc, Batavia, Illinois), and the treatment group received the corn syrup mixture combined with 5 g WLS herbal powder (Stomach Happy; Jing Tang Herbal, Inc, Reddick, Florida) administered by syringe twice daily. Between treatment periods, the horses were turned out to pasture during the day, with access to free choice hay at night, as a 4‐week washout period.

Gastroscopic examinations were performed on days 0, at the end of the feed‐deprivation period (day 6), and at the end of the fourth week of treatment (day 34) for each period. Before gastroscopy, feed and water were withheld for 24 and 4 hours, respectively, to improve gastric visualization. The horses were sedated with a combination of IV detomidine hydrochloride (Dormosedan; Zoetis, Parsippany, New Jersey) and butorphanol tartrate (Torbugesic; Zoetis; 0.01 mg/kg of each) for the examination, and a 3‐m endoscope (Storz, 3‐m endosope, model #60130NKSK; Karl Storz Veterinary Endoscopy‐America, Inc, Goleta, California) was used for endoscopy. The stomach was insufflated with room air to allow for visualization. After insufflation, a minimum of 60 mL of gastric fluid was aspirated from a level halfway between the surface of the fluid and the ventral stomach wall (approximately 2‐3 cm below the gastric fluid line) through the endoscope biopsy channel using a portable suction pump (EasyVac aspirator, model PM60; Precision Medical, Inc., Northampton, Pennsylvania). Gastric juice pH was measured immediately with a portable pH meter (LAQUAtwin compact pH meter, B‐213; Horiba, Ltd, Kyoto, Japan) with an attached electrode. The pH meter was calibrated with a set of standard (pH 4.0, 7.0) solutions provided by the manufacturer before the beginning of each collection period. Scoring was performed once the stomach was insufflated to the point that the rugae were no longer apparent. To improve visualization, the nonglandular mucosa was rinsed of adhered ingesta with tap water introduced through the biopsy channel. The stomach was evaluated, and a gastric ulcer score given (range 0‐4 for number of ulcers, range 0‐5 for severity of ulcers) for the nonglandular squamous mucosa, based on a previously established scoring system for horses.^14^, ^21^ (Table 1). Scores were assigned by 3 evaluators, 2 during the endoscopy and 1 from recorded images, who were blinded to the treatment group.

### 2.2 Statistical analysis

Data were collated and collected using a commercially available software program (Excel, Microsoft Windows 2016; Microsoft, Inc, Redmond, Washington), and the median scores from the 3 evaluators were used to assess the gastric ulcer number and severity scores. The gastric pH values were treated as continuous variables and were presented as mean and standard deviation. The interrater reliability was assessed using a kappa statistic as well as a Kendall's coefficient of concordance to evaluate the agreement between evaluators and provide the associated standard error with SAS MACRO MAGREE.^23^, ^24^ Because 10 horses were repeatedly used for 2 treatments and repeatedly measured at pretreatment, baseline and posttreatment, the gastric fluid pH value was analyzed with a linear mixed‐effect model using SAS PROC GLIMMIX. The gastric ulcer number score and ulcer severity scores were analyzed with generalized estimated equations (GEE) with repeated measures using SAS PROC GENMOD. Horses were treated as the random effect, and treatment, time point, and treatment × time point were treated as the fixed effects. Statistical analyses were performed using SAS software version 9.4 (SAS Institute, Cary, North Carolina). All *P* values were 2‐sided, and *P* < .05 was used to indicate statistical significance.

## 3 RESULTS

Physical examinations were within normal limits at the time of enrollment and over the duration of both treatment periods. All mares enrolled gained weight over the course of the study, and feed was not adjusted for any mare during the trial. The corn syrup mixture as well as the WLS herbal formula was readily accepted by all horses in the study. One horse in the treated group developed mild signs of colic, consisting of flank watching, during the first period of the study. Physical examination noted no changes in vital parameters, and clinical signs resolved without treatment. A second horse on the control group was treated for a corneal abrasion with 2 doses of flunixin meglumine (1.1 mg/kg, IV), topical atropine, and topical application of an antibiotic ointment on the last 4 days of the first period. No horse developed diarrhea or anorexia during the duration of the study.

### 3.1 Nonglandular gastric ulcer scores

The resulting interrater reliability was excellent for both ulcer number (overall Kappa = 0.65; Kendall's coefficient of concordance = 0.95) and ulcer severity scores (overall Kappa = 0.63, Kendall's coefficient of concordance = 0.92), indicating that the evaluators had a good‐to‐high degree of agreement and suggesting that ulcer scores were rated similarly.^25^, ^26^

**Figure 1:**
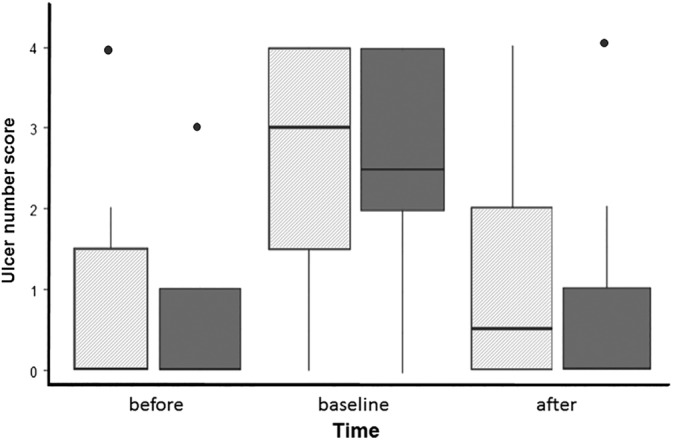
Boxplot (median, 25% and 75% quartiles, and range) showing the effect of treatment with a placebo or the herbal supplement Wei Le San (WLS) on median ulcer number score over time. The time point of each endoscopy during the treatment period (pretreatment (before), baseline at 6 days, and after treatment [after, 35 days]) is provided on the x‐axis and the gastric ulcer number score is on the y‐axis. The hatched boxes represent placebo; solid boxes represent the treatment group. There was no difference in gastric ulcer number scores between the placebo and the herbal treatment (*P* = .81)

**Figure 2:**
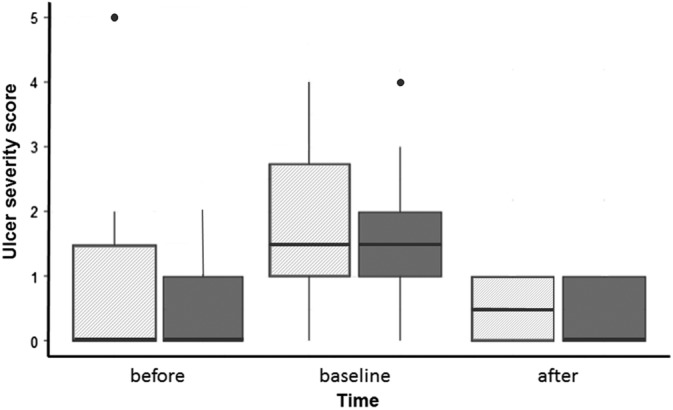
Boxplot (median, 25% and 75% quartiles, and range) showing the effect of treatment with a placebo or the herbal supplement Wei Le San (WLS) on median ulcer severity score over time. The time point of each endoscopy during the treatment period (pretreatment (before), baseline at 6 days, and after treatment [after, 35 days]) is provided on the x‐axis and the gastric severity score is on the y‐axis. The hatched boxes represent placebo; solid boxes represent the treatment group. There was no difference in gastric ulcer severity scores between the placebo and the herbal treatment (*P* = .85)

Applying the GEE procedure for ordinal data, there was no significant treatment × time point interaction; therefore, only treatment and time were used as the fixed effects. Before the start of the study, 4 of 10 horses in the treatment group and 3 of 10 horses in the control group had gastric ulcers. On day 6 (after the week of feed deprivation), 8 horses in the control group and 9 horses in the treatment group demonstrated squamous gastric ulceration. After completing the 4 weeks of treatment, 5 of 10 control horses still had gastric ulceration, compared to 4 of 10 in the treatment group on day 34. The probability of higher ulcer number and ulcer severity scores (*P* = .81 and *P* = .85, respectively) was not different between horses in the treatment and control groups of the study at the end of the treatment periods, but both scores increased significantly at day 6 after 1 week of feed deprivation for both groups (*P* < .001). (Figures 1 and 2) However, after the 4 weeks of washout between the 2 study periods, gastric ulceration resolved in all horses. Scores at the start of the second period were not different than the rankings on day 0 of the first study period (ulcer number scores: *P* = .95; ulcer severity scores: *P* = .43).

### 3.2 Gastric fluid pH

**Figure 3:**
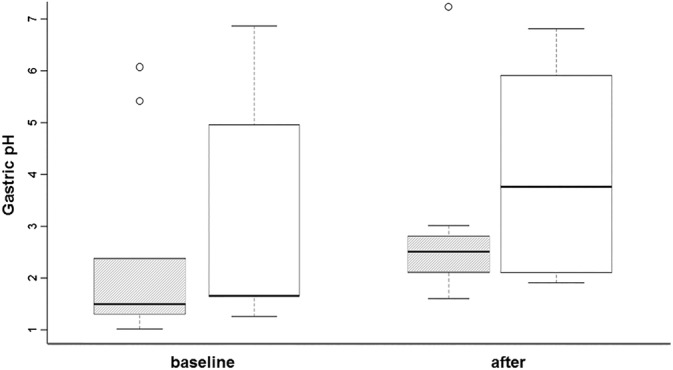
Boxplot (median, 25% and 75% quartiles, and range) showing the effect of treatment with a placebo on gastric fluid pH over time. The time point of each endoscopic sample during the treatment period (baseline at 6 days, and after treatment at 35 days) during the treatment period is provided on the x‐axis, and the pH is on the y‐axis. The hatched boxes represent placebo; solid boxes represent the treatment group. There was no difference in gastric pH over the course of treatment between control and herbal treatment groups compared to baseline values (*P* = .46)

Gastric fluid pH was variable throughout the study, ranging from 1.0 to 7.2. On day 6 before administration of the treatments, the median gastric fluid pH was 1.6 (range 1.2‐5.5) for the horses treated with WLS, and 1.5 (range, 1.2‐5.5) in the untreated controls. After 4 weeks of treatment, the median gastric fluid pH increased marginally and was 2.1 (range, 1.9‐4.1) for the horses treated with WLS and 2.8 (range, 1.6‐7.2) in the untreated controls. There was no difference noted after 4 weeks of treatment with WLS or the untreated control compared to baseline (*P* = .46; Figure 3).

## 4 DISCUSSION

Administration of a Chinese herbal formulation in this experimental model was not effective in reducing squamous gastric ulcer severity in horses caused by intermittent feed deprivation. Glandular ulceration was not observed in horses evaluated in this study, even in those with severe nonglandular ulceration scores; therefore, this type of ulcer could not be evaluated by this protocol. The administration of the herbal supplement was not associated with linked to changes in gastric fluid pH.

The proprietary veterinary formulation WLS contains herbs claimed to “rebalance the liver and stomach, nourish Yin, and move Qi to resolve stagnation and relieve pain.”^17^ It is intended for administration to horses with mild to moderate gastric ulceration.^16^, ^17^ The formula contains the following herbs: *Paeonia lactiflora* (Bai Shao Yao) root with the bark removed, *Atractylodes lancea* (Cang Zhu) rhizome, dried rind of the mature *Citrus reticulata* (Chen Pi) fruit, *Angelica sinensis* (Dang Gui) root, *Trichosanthes* (Gua Lou) fruit, *Glycyrrhiza uralensis* (Gan Cao) root and rhizome, *Taraxacum mongolicum* (Pu Gong Ying) in its entirety, the immature fruit of the *Poncirus trifoliata* (Zhi Shi), and *Bupleurum chinense* (Chai Hu) root. Further investigations are needed to identify any actions of the WLS herbal formula and its constituents on gastric ulcer healing and outcomes.

A limitation of this study was that although the intermittent feed deprivation model was successful in inducing nonglandular gastric ulcers, not all horses responded to the protocol. This was not unexpected, based on a previous work that used a similar model.^22^ One difference within our study was that the horses were allowed to remain on free choice hay between periods of intermittent fasting, which followed the original protocol that was designed to produce consistent ulceration of the squamous mucosa.^27^ It is likely that if we had used a pelleted feed or reduced the hay intake to <2% of body weight, the squamous ulcers after feed deprivation could have been more severe.

Gastric fluid pH was variable and was not significantly different between WLS‐treated horses and the untreated controls. Although a single gastric fluid sample is not as likely to note changes in pH that occur over a 24‐hour period, continuous monitoring might have been able to better identify change in gastric fluid pH.^28^, ^29^ However, the sampling protocol was uniform with regards to fluid depth location and obtained in fasted horses where the pH should have been at its nadir due to the absence of buffering feed and lower levels of saliva production.^30^

An additional limitation of the pH measurements in this study was the use of alpha‐2 agonists and butorphanol for the gastroscopy procedure, which could have resulted in reduced gastrointestinal motility.^31^ Although these medications are known to reset the migrating motility complex, the effects of butorphanol are believed to be minimal, whereas those of detomidine can markedly reduce duodenal motility.^32^, ^33^ The time frame from sedation to sample collection was minimized (<10 minutes), in an effort to reduce the influence of duodenal stasis on gastric contents.

The results of this study indicate that the Chinese herbal WLS was unsuccessful in reducing the severity of experimentally induced, nonglandular gastric ulcers in horses exposed to intermittent feeding compared to a placebo control. It is possible that the model used to produce gastric ulceration in these horses was not severe enough to produce a significant difference between the groups on a free choice hay diet by the end of the study period, leading to a type II error. It is also likely that the diet could have contributed to the improvement in gastric ulcer scores in both groups, as a hay diet is known to increase saliva production that can buffer stomach contents and reduce acid exposure.^34^ Further investigations could involve a feed deprivation challenge at the end of the administration period, to determine the herbal supplement's effect in the face of a feed deprivation challenge. Additional evaluation is needed in a larger number of horses, and in clinical cases, where feeding protocols and husbandry practices are not as tightly controlled.

## CONFLICT OF INTEREST DECLARATION

Authors declare no conflict of interest.

## OFF‐LABEL ANTIMICROBIAL DECLARATION

Authors declare no off‐label use of antimicrobials.

## INSTITUTIONAL ANIMAL CARE AND USE COMMITTEE (IACUC) OR OTHER APPROVAL DECLARATION

This study was approved by the University of Wisconsin Institutional Care and Use Committee, Protocol #V005953.

## HUMAN ETHICS APPROVAL DECLARATION

Authors declare human ethics approval was not needed for this study.
